# Non operative management of traumatic esophageal perforation leading to esophagocutaneous fistula in pediatric age group: review and case report

**DOI:** 10.1186/s13017-015-0012-y

**Published:** 2015-04-02

**Authors:** Biplab Mishra, Saurabh Singhal, Divya Aggarwal, Nitesh Kumar, Subodh Kumar

**Affiliations:** Jai Prakash Narayan Apex Trauma Center, All India Institute of Medical Sciences, New Delhi, India; All India Institute of Medical Sciences, New Delhi, India; University College of Medical Sciences, New Delhi, India

**Keywords:** Esophageal perforation, Perforation, Pediatric, Traumatic, Non-operative, Conservative, Protocol, Thoracic, Iatrogenic

## Abstract

Management of delayed presenting esophageal perforations has long been a topic of debate. Most authors consider definitive surgery being the management of choice. Management, however, differs in pediatric patients in consideration with better healing of younger tissues. We extensively review the role of aggressive non-operative management in pediatric esophageal perforations, especially with delayed presentation and exemplify with case of a young boy with esophageal perforation and esophago-cutaneous fistula. We also lay down the protocol to manage such patients based on our institutional recommendations.

## Background

Management of esophageal perforations (EPs) has long been a topic of debate. The management protocols are chiefly governed by symptom severity, perforation site, time elapsed since perforation and cause of perforation. Esophageal perforations can be iatrogenic, traumatic, spontaneous or following forceful vomiting. Penetrating non-iatrogenic EP is a rare, life-threatening condition [1-4]. Surgical interventions including primary repair with tissue reinforcement or resection-reconstruction have long been the preferred approach [4]. Non operative management is generally advocated in contained leaks, iatrogenic injuries and hemodynamically stable patients. It is not recommended in delayed EPs (presenting after 24 hours) [5]. We review the literature on the role of non-operative management in EPs and describe management of a pediatric case with delayed traumatic thoracic EP with esophago-cutaneous fistula.

### Case presentation

An 11 year old male with alleged history of penetrating trauma to lower chest presented to a local community hospital. While playing at a construction site, the child fell on a sharp iron rod which inflicted the injury. He was managed with fluid resuscitation followed by removal of the rod through the entry wound. The wound was thoroughly irrigated and dressed. No other surgical intervention was done. On day 1, the child developed lower chest pain, dyspnea and low grade fever. Chest x-ray revealed right sided moderate hydropneumothorax for which intercostal drain (ICD) was placed. No further imaging studies were done. Child was kept nil per oral (NPO) with intravenous (IV) fluids and nutritional supplements for first two days; analgesics and IV amoxicillin-clavulanate were given for five days. No naso-gastric (NG) tube insertion was done during the hospital stay. There were no further fever episodes. Local wound care and regular dressings were done.

Child was allowed oral liquids on day 4. Ingested liquids were found to be coming out of the entry wound. There was no associated chest pain or dysphagia. Patient was again kept NPO for another ten days with repeat trials of oral feeds thrice in this duration. On similar observation, possibility of esophageal perforation with esophago-cutaneous fistula was made and feeding gastrostomy (FG) was done for enteral nutrition. Patient was then referred to our tertiary care level-I trauma centre.

Child presented to our emergency department on day 13 following injury. He was lethargic and malnourished with a GCS of 15/15, though did not appear to be in any acute distress. Airway was patent, with reduced air entry and crepitation in right lower zone and saturation >97% on room air. Chest compression test was negative. He was afebrile with a pulse rate of 104 per minute and blood pressure of 102/60 mmHg. Capillary filling time was normal. Child weighed 10 kg with height of 98 cm. He was afebrile to touch.

On examination, a 3×3 cm entry wound was noted 2 cm lateral to the right border of sternum, in 6^th^ intercostal space, about 3.5 cm below right nipple. Wound was healthy with granulation tissue and sero-mucoid discharge. There was 24 Fr ICD in situ in right 4^th^ intercostal space and a feeding gastrostomy in place. Total ISS score and Braden score at presentation were 18 and 19 respectively.

**Figure 1:**
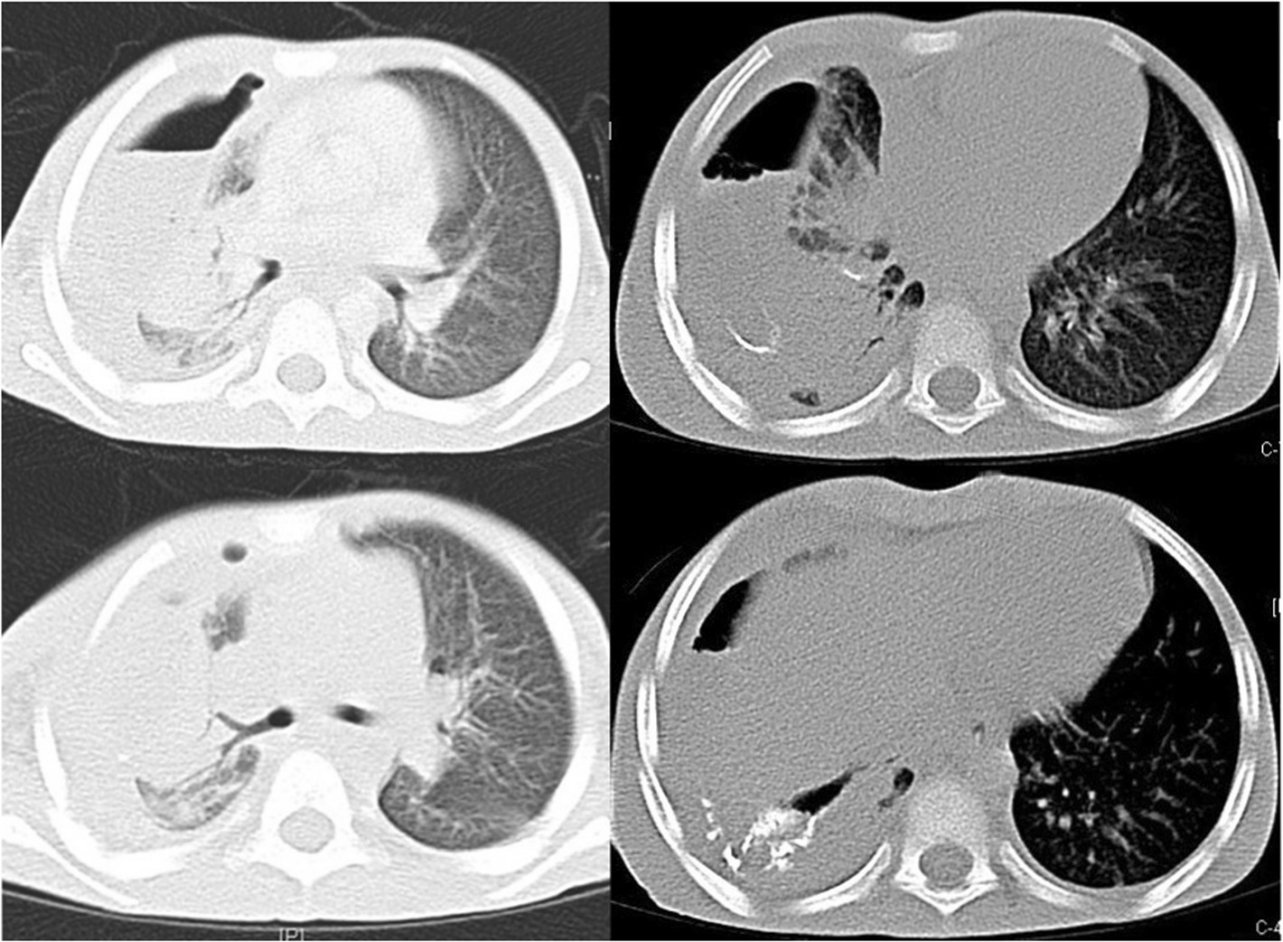
**CECT chest showing contrast leak from thoracic esophagus with pooling of contrast in right pleural cavity.** Lung consolidation may be appreciated.

Chest roentgenogram revealed right lower lobe consolidation and right sided pleural effusion with ICD in situ. A contrast enhanced CT scan (CECT) of chest and abdomen was done with additional non-ionic contrast given orally (Figure 1). It revealed right sided hydropneumothorax with contrast leak from thoracic esophagus, pooling of contrast in right pleural cavity, draining through entry wound and ICD, and right sided mid and lower lobe lung contusions with consolidation of right lower lobe. Left lung was healthy with no significant radiologic abnormalities detected. There was visible contrast leak from the skin wound as well.

Patient was admitted and managed conservatively with IV fluids, IV antibiotics (cefoperazone-sulbactam for 10 days and metronidazole for 6 days), adequate wound care and nutritional care. He was kept NPO on parenteral nutrition with vitamin K supplements. No NG tube insertion was done. FG feeding, alongwith electrolyte and vitamin C supplements, was initiated on day 2 of admission at 30 mL/hour and gradually increased to 50 ml/hour as it was well tolerated. ICD was kept on under water seal drainage. Patient’s progress records have been charted in Table 1.

**Table 1:** **Progress chart of patient during in-hospital stay**

	**Presentation**	**Day 2 of admission**	**Day 10 of admission**	**Discharge**
Weight (kg)	10	10.3	12.8	13.2
Pulse rate (per minute)	104	92	94	91
Temperature (°F)	99.1	97.4	98.1	98.6
Braden risk	19	20	20	21
Hemogram				
Hb (gm%)	9.5	10.1	11.5	10.9
Hct (%)	27.3	32.7	37	36.8
Plt (per cumm)	567,000	805,000	796,000	512,000
TLC (per cumm)	15,500	14,100	11,800	9,800
Blood biochemistry				
U/Cr/Na/K	15/0.3/137/4.1	15/0.3/135/4.8	26/0.2/137/5.5	24/0.4/133/4.2
Serum Protein	4.2	4.3	6.6	6.8
Serum Albumin	2.3	2.6	3.3	3.5

Hb- Haemoglobin; Hct- Haematocrit; Plt- Platelet count; TLC- Total leucocyte count; U- Urea; Cr- Creatinine; Na- Sodium; K- Potassium.

**Figure 2:**
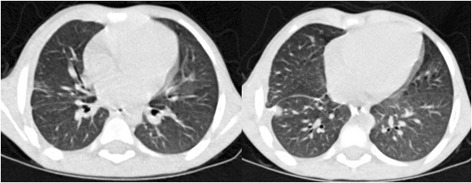
**Repeat CECT chest on day 20 of admission revealed no contrast leak.**

On day 20 of admission, ICD removal was done as drain output was minimal (serous) and ICD fluid cultures were consistently negative. Repeat CECT chest with oral contrast revealed no leak (Figure 2). Full oral diet was initiated.

Child was discharged on day 22 of admission after removing FG. On discharge, child was in good health, accepting orally with stable vitals, bilaterally clear chest and soft, non-tender abdomen. He gained 3.2 kg during hospital stay and total leucocyte count fell from 15,500/cumm to 9,800/cumm. Braden risk score remained above 19 throughout hospital stay. Wound healed with secondary intention.

**Figure 3:**
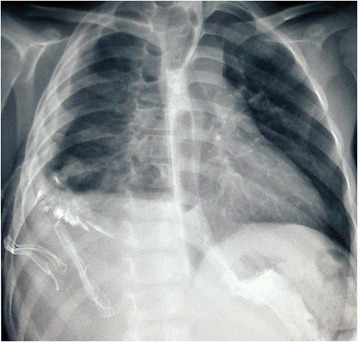
**Barium swallow at 2 months follow-up revealed no contrast leak.**

Repeat barium swallow on two month follow-up revealed no leak (Figure 3). Chest x-ray revealed clear lung fields bilaterally. Patient is doing fine on 18 month follow-up, with weight and height appropriate for age, and is accepting oral feeds. There are no respiratory symptoms, dysphagia or chest pain. Scar at wound site is healthy.

## Review and discussion

Esophageal perforation (EP); traumatic, iatrogenic or due to any other cause; has long been a dreaded condition with high morbidity and mortality rates. The first account of EP comes from late 18^th^ Century as described by Boerhaave [6]. First pediatric perforation was described by Fryfogle in 1952 [7].

EP is a life threatening condition associated with mortality rates reaching upto 20-50% [7-10]. Contamination with oral and gastro-intestinal contents can cause mediastinitis and generalized sepsis leading to multi-organ dysfunction and death [11]. Delay in diagnosis is not uncommon owing to the more common differentials with similar presentation and is dreadful, unless there is a temporal relationship present with esophageal instrumentation or trauma to have high suspicion of EP [12]. With advent of esophago-gastric instrumentation, iatrogenic causes have replaced the other causes as the most common etiology. Traumatic perforations are very rare but demand a high index of suspicion owing to their high morbidity and mortality [4] (Table 2).

**Table 2:** **Aetiology of esophageal perforation (in descending order of incidence) [** 4,14-16]

**Children**	**Adults**
1) Iatrogenic (diagnostic or therapeutic instrumentation)	1) Iatrogenic (diagnostic or therapeutic instrumentation)
2) Lye burns	2) Spontaneous (Boerhaave’s syndrome)
3) Direct/Indirect trauma	3) Foreign bodies
4) Foreign bodies	4) Penetrating trauma (m.c.- gunshot)
5) Operative procedures in the area	5) Malignant perforations
6) Idiopathic	6) Operative injury
	7) Idiopathic

m.c. – most common.

Historically, early surgical intervention (within 24 hours of presentation), with intent of definitive repair, used to be the mainstay of treatment owing to the reported mortality rates as high as 69% in patients managed non-operatively or in whom surgeries were delayed. Early surgical interventions were considered to bring down mortality rates to less than half [13]. Primary surgery had since been considered the management of choice for EP in adults and most children except for few early presenting cases [13-18]. Okanta et al [5] reviewed seven major studies describing management of delayed benign esophageal perforations and concluded esophagectomy as better management approach compared with primary repair and conservative management. Their review, however, mostly included retrospective studies, lacked randomized controlled trials and adequate follow-up and did not differentiate between mortalities for early and delayed EPs in many of the studies.

First published account of successful non operative management for EPs came from work of Mengoli and Klassen in 1965. They achieved mortality rates of about 6% in 18 cases of iatrogenic esophageal perforations (following diagnostic or therapeutic esophagoscopy) managed conservatively. Two-third of their patients had perforation in distal third of the esophagus. They relied on massive use of antibiotics, nasoesophageal suction and intercostal drainage [19-21]. Brinster et al [4] reviewed various series published between 1990 and 2003 for management options for EPs and concluded a total mortality of 18% with any kind of treatment. Mortality with non-operative management (17%) was slightly higher than the primary repair (12%) whereas it was much higher with drainage (36%) and exclusion (24%).

Increasing incidence of iatrogenic injuries, which are earlier diagnosed and are associated with less mediastinal contamination, are ideal for non-operative management. Less contamination is due to nil per oral status of patient prior to endoscopic procedures and injuries mostly being limited. Traumatic injuries have lesser evidence but yet have been proven to show successful healing with the latter, as was in our patient. Not to forget the younger age, which has a positive impact in healing of tissues.

Thoracic EPs are more amenable to successful non-operative management owing to ease of pleural drainage for esophageal leaks [14]. With adequate pleural toilet, proper antibiotic coverage and nutritional support, the thoracic esophageal perforations as well as esophago-cutaneous fistulas heal spontaneously, just like any other gastrointestinal fistulas [15].

EP in children have special relevance in view of inability of very young children to present with early signs and symptoms. Most perforations in pediatric age group are iatrogenic following upper airway or esophageal corrosive esophageal injuries [7] (Table 2). Children developing chest or abdominal pain, nausea, dyspnea, fever, leucocytosis, subcutaneous emphysema and other signs and symptoms following esophageal instrumentation or trauma to lower neck, chest or upper abdomen should be dealt with high index of suspicion [4,12,22]. Early diagnosis is vital. Prognosis is better with diagnosis within 24 hours of perforation. Chest X-rays, water soluble or non-ionic contrast studies of esophagus and contrast enhanced CT scan with oral contrast should be utilised for early and accurate diagnosis [4]. Endoscopy may be combined with contrast studies for accurate diagnosis and can play a therapeutic role in the same sitting. Raised drain amylase is another sensitive but non-specific indicator of esophageal injury [22]. Favourable prognostic factors are listed in Table 3.

**Table 3:** Favourable prognostic predictors after EP* [ 17,39]

1.	Early diagnosis and treatment
2.	Iatrogenic origin
3.	Young age
4.	Absence of concomitant esophageal disease
5.	Benign perforations
6.	Absence of co-morbidities
7.	Good nutritional and hemodynamic status
8.	Site- Cervical > Thoracic (Abdominal EP generally has poor outcome)
9.	Sharp penetrating injuries better than blunt and thermal puncture (gunshot) injuries

*Apply to both operative and non-operative management.

Non-operative approach to pediatric EPs stem from the unparalleled healing capacity of tissues at younger age [23]. Martinez et al [13] published an elaborate case series of non-operative management of EPs in children. They successfully managed 17 of 18 pediatric cases of thoracic esophageal perforations. They had 100% survival rate with only one patient developing long term esophageal stricture requiring dilatation. Their results emphasize the importance of non-operative management in pediatric age-group. Children with caustic injury are prone to iatrogenic esophageal injuries during endoscopic balloon dilatation for strictures. A conservative approach with or without cervical esophagostomy and gastrostomy has been found to be adequate in such patients. Resection anastomosis and colonic interpositions may be considered in patients with long segment strictures following perforation [24]. Delayed EPs, extensive involvements and esophago-cutaneous fistulas, which are relatively contraindicate conservative management, can still be managed successfully by active and aggressive non-operative approach in children.

A recently published position paper on esophageal injuries recommends non-operative management to be done in hemodynamically stable patients with small perforations presenting within 48 hours of injury [22]. We agree with the recommendations and emphasize the importance of aggressive conservative management in pediatric population (as per our protocol flowchart).

Neonatal esophageal perforation is mostly seen in premature new-borns with history of multiple attempts at intubation or forceful oropharyngeal suctioning. Various authors have shown the successful non-operative approach with minimal surgical interventions for such patients [25,26].

Adequate nutritional support is of prime concern in children. Enteral feeding is always considered superior to prolonged parenteral support, which has its own drawbacks. Feeding gastrostomy and jejunostomy are considered limited surgical interventions and should be included in non-operative approach to pediatric EPs. Apart from providing nutritional support, they help in preventing retrograde contamination of mediastinum with gastric secretions [20].

To prevent mediastinal contamination, nasogastric drainage is suggested and practised by some physicians, though its role has long been debated. While many authors include it in the non-operative regime [27], Cameron et al. achieved uncomplicated spontaneous closure of esophageal leaks in all eight patients without even pleural drainage, seven of whom did not undergo nasogastric drainage as well. They claim that latter only increases gastro-esophageal reflux which will further aggravate mediastinal contamination [28,29]. We do not recommend nasogastric drainage in our protocol, especially in pediatric age group. Our patient, without nasogastric drainage, achieved successful outcome which further affirms our recommendations.

There are some recent studies addressing uses of endoscopically placed self-expandable metallic stents with or without chest drainage in patients with esophageal perforations and post-operative esophageal anastomotic leaks [8,30-34]. However, none of the studies have sufficiently large sample size and long term follow-ups to look at possibility of esophageal strictures associated with metallic stents in situ [8,24,35-37]. Also, there are very few cases among pediatric age group. While stent placement can act as a bridging option to definitive surgeries like esophagectomy and colonic interposition, however, there are reports of esophageal stents themselves causing esophageal injury [8,22,24]. Displaced stents may also be of concern in younger children. We recommend more studies on their usage. We, currently, do not include esophageal stent placement in the management protocol of pediatric or adult esophageal perforations at our institution.

Use of endoscopically placed clips and endoscopic vacuum sponge are the other newer modalities being introduced with promising results. The adequately powered randomised and blinded trials are required to prove their efficacy in children [38].

**Figure 4:**
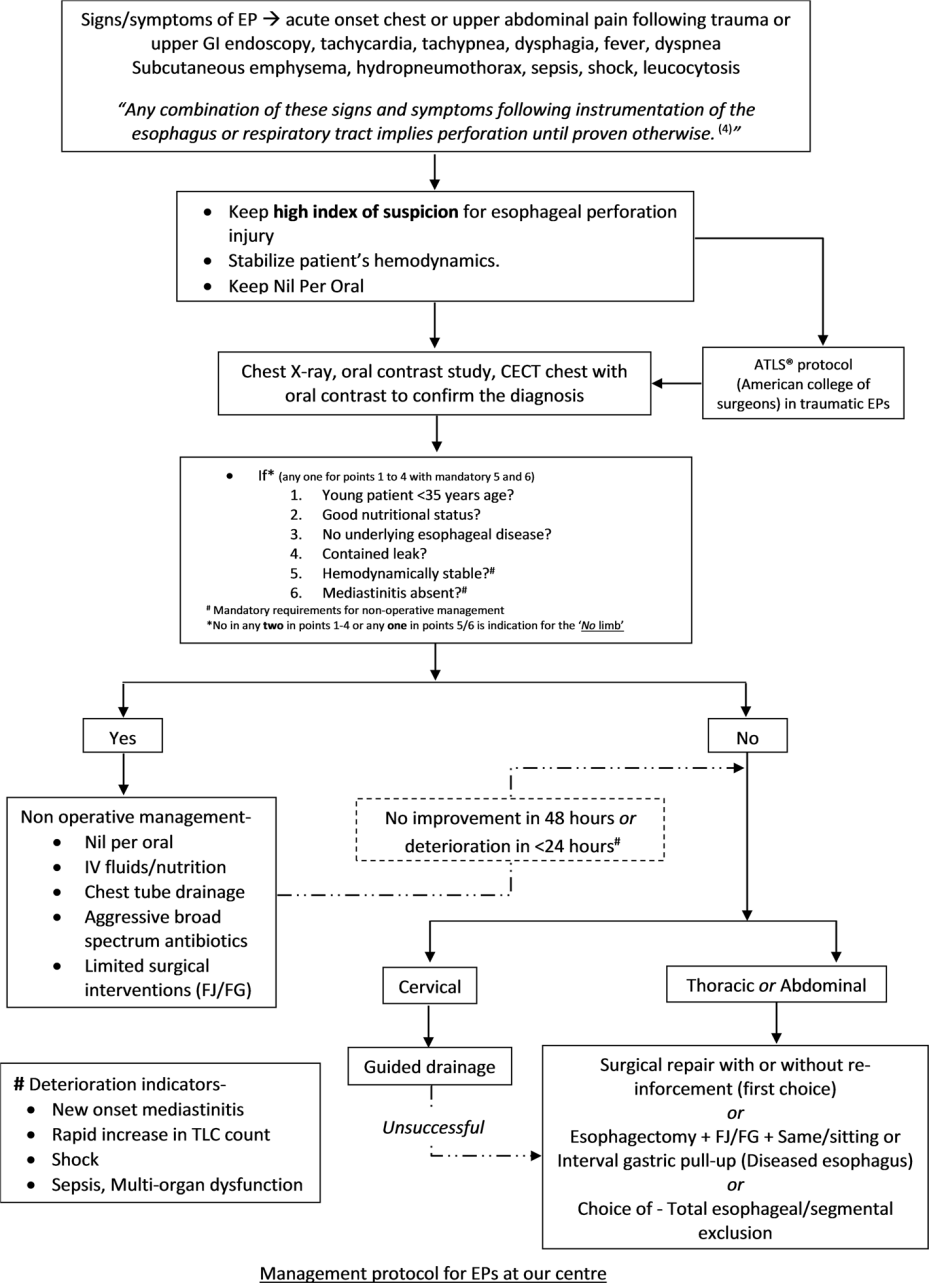
**The management protocol for pediatric esophageal perforations at our level I trauma center.**

Overall, non-operative management protocols, with advent of early diagnostic modalities and close monitoring in delayed presentations, are useful and should be implemented in carefully chosen patients. Our protocol for management of esophageal perforations is shown in the form of a flowchart in Figure 4. Non-operative management protocol has been described in Table 4.

**Table 4:** **Non-operative management protocol for pediatric esophageal perforations (at our centre)**

**Intervention**		**Significance**
1)	Nil per oral (minimum of 7–10 days)	+++
2)	Adequate enteral/parenteral hyperalimentation	+++
3)	Aggressive broad spectrum antibiotic therapy (minimum 7 days)	+++
4)	Early limited surgical interventions (gastrostomy/jejunostomy)	+
5)	Chest drainage with wide bore intercostal drain	++
6)	Nasogastric suction/drainage	+/−
7)	Intravenous proton pump inhibitors (minimum 7 days)	+/−

## Conclusion

EPs are rare in children and traumatic EPs are even rarer. We conclude that they can successfully be managed by an active and aggressive non-operative approach. A good antibiotic coverage, nutritional support, downstream drainage of leaks via intercostal drains and occasional need for limited surgical interventions as gastrostomy and jejunostomy are vital and may even be employed in extensive and delayed EPs. Authors still recommend attending physician’s discretion in planning the management and deciding for early definite surgical interventions depending on individual presentations.

### Consent

Informed and written consent was taken from the patient’s parents to publish this case report, investigation reports and images.

## Abbreviations

EP: Esophageal Perforation
CECT: Contrast enhanced computed tomography
ICD: Intercostal drain
IV: Intravenous
FG: Feeding gastrostomy
FJ: Feeding jejunostomy
